# Predialysis predictors for identifying patients requiring dialysis at a higher glomerular filtration rate

**DOI:** 10.1080/0886022X.2021.1940202

**Published:** 2021-07-05

**Authors:** Junseok Jeon, Hye Ryoun Jang, Wooseong Huh, Yoon-Goo Kim, Dae Joong Kim, Jung Eun Lee

**Affiliations:** Division of Nephrology, Department of Medicine, Samsung Medical Center, Sungkyunkwan University School of Medicine, Seoul, Korea

**Keywords:** Maintenance hemodialysis, end-stage kidney disease, early initiation of dialysis, unplanned dialysis, predialysis predictors

## Abstract

**Background:**

Current evidence suggests that the initiation of maintenance hemodialysis should not be based on a specific glomerular filtration rate (GFR) but on symptoms or signs attributable to kidney disease. However, it is difficult to predict the time point at which overt uremic syndrome develops in individuals. The estimated GFR is poorly correlated with occurrence of uremic symptoms, and some patients require dialysis at a higher eGFR than others. In this case, patients are more likely to be improperly prepared for dialysis. We investigated the predialysis characteristics of patients who require dialysis at a higher eGFR.

**Methods:**

A total of 453 incident dialysis patients being monitored by a nephrologist from January 2013 to December 2018 were included. The predialysis characteristics when eGFR decreased to 20 mL/min/1.73 m^2^ were obtained.

**Results:**

The mean age was 61 years, and 65.7% were men. Overall, the median eGFR at the first dialysis was 5.8 (interquartile range 4.6–7.3) mL/min/1.73 m^2^ and initiation of dialysis at the first quintile (≥7.8 mL/min/1.73 m^2^) was defined as ‘early initiation of dialysis’ Among the predialysis characteristics, heart failure (adjusted odds ratio 3.68; 95% confidence interval, 1.59–8.03), serum albumin <4.0 mg/dL (2.22; 1.30–3.77), blood urea nitrogen (BUN)/creatinine (Cr) ratio >15 mg/mg (1.92, 1.16–3.18), and hyperuricemia (1.84; 1.05–3.23) were independent predictors of early initiation. Diabetes mellitus and the causes of kidney disease were not independent predictors of early initiation. The early initiation group was less likely to initiate dialysis with a permanent vascular access than the late initiation group.

**Conclusions:**

For patients with heart failure, low serum albumin level, high BUN/Cr ratio, or hyperuricemia, clinicians can provide predialysis counseling in advance and consider early creation of vascular access.

## Introduction

Chronic kidney disease (CKD) has a high worldwide prevalence and is a major burden to public health [1]. Despite the advances in the management of CKD and the widespread application of kidney transplantation, the number of incident patients who require renal replacement therapy (RRT) has remained constant over the last decade. It has been estimated that approximately 2.618 million patients undergo RRT worldwide [2]. For a smooth transition from CKD to end-stage kidney disease (ESKD), provision of balanced information on RRT modality and timely preparation of dialysis access are essential during the pre-ESKD period. Moreover, estimation of the best time for initiating RRT should precede the application of optimal pre-ESKD care.

The optimal timing of dialysis initiation and its impact on long-term outcome have been controversial [3–6]. A decade ago, clinical practice guidelines suggested cutoff levels of 15 mL/min/1.73 m^2^ for glomerular filtration rate (GFR), for initiating dialysis, even without symptoms or signs attributable to kidney failure [7]. However, growing evidence, including that from the IDEAL (Initiating Dialysis Early and Late) study, indicates that early initiation of dialysis in asymptomatic patients has no survival benefit [8]. Consequently, the updated policy in the 2012 KDIGO (Kidney Disease: Improving Global Outcomes guideline) recommends initiating dialysis on the basis of uremic symptoms or complications rather than the GFR value [9].

However, it remains uncertain how to predict the time point at which overt uremic syndrome develops in individuals. The estimated GFR (eGFR), generally used as an indicator of residual kidney function, is poorly correlated with the concentration of uremic toxins in advanced CKD, and some patients initiate dialysis at a higher eGFR than others. If dialysis is initiated at high eGFR, patients would be less prepared for dialysis, making the initiation process psychologically more challenging and increasing the likelihood of starting dialysis through intravascular catheters. Therefore, it is crucial to identify patients who require dialysis at a higher eGFR, as earlier applications of pre-ESKD care are also needed for those individuals. In this retrospective study, we aimed to investigate the predialysis characteristics of patients who require dialysis at a higher eGFR in real-world settings.

## Materials and methods

### Study design and population

We screened a total of 688 adult (age ≥18 years) patients who received pre-ESKD care for ≥6 months and initiated chronic hemodialysis from January 2013 to December 2018 in Samsung Medical Center, a 1989-bed, tertiary teaching hospital. A total of 453 patients were finally included in the study after the exclusion of those who met the following criteria: insufficient data (*n* = 169), eGFR >30 mL/min/1.73 m^2^ within 1 year before dialysis initiation (*n* = 20), and previous kidney transplantation (*n* = 46).

The study was approved by the Institutional Review Board of Samsung Medical Center in compliance with the Declaration of Helsinki (approval no. 2019-04-128). The Institutional Review Board waived the requirement for informed consent because data were retrospectively collected.

### Measurements

We extracted data from electronic medical records. Predialysis was defined as the time point immediately before reaching an eGFR of <20 mL/min/1.73 m^2^ [10]. The predialysis demographic and clinical characteristics included age, sex, body mass index, diabetes mellitus (DM), hypertension, ischemic heart disease, heart failure, malignancy, and use of loop diuretics, angiotensin-converting enzyme inhibitors/angiotensin receptor blockers, or statin (HMG-CoA reductase inhibitors). The presence of comorbidities, including heart failure, was defined as the presence of the corresponding diagnostic code from 9 months before the index date to 3 months later. Predialysis laboratory data included serum creatinine (Cr), blood urea nitrogen (BUN), hemoglobin, serum albumin, total carbon dioxide, uric acid, calcium, phosphate, N-terminal prohormone of brain natriuretic peptide, and urine protein/creatinine ratio. eGFR was calculated using the Chronic Kidney Disease Epidemiology Collaboration (CKD-EPI) equation [11]. Laboratory data obtained at the initiation of dialysis were also retrieved. Anemia was defined as hemoglobin level <13.5 mg/dL in men and <12 mg/dL in women. Hyperuricemia was defined as serum uric acid level >7.0 mg/dL in men and >6.0 mg/dL in women. Serum corrected calcium was calculated as follow: corrected calcium (mg/dL)=serum calcium (mg/dL) × 0.8 × (4 – serum albumin [mg/dL]).

### Definitions

Early initiation of dialysis was defined as starting dialysis at eGFR ≥7.8 mL/min/1.73 m^2^ based on the upper 20% of eGFR at the initiation of dialysis [12]. Unplanned dialysis was defined as not creating an arteriovenous fistula/arteriovenous graft at least 1 month before the initiation of dialysis.

### Statistical analyses

Continuous variables are presented as mean ± standard deviation or median (interquartile range), and categorical variables are presented as number (percentage). For group comparisons of continuous variables, an independent t-test or Mann-Whitney U-test was used according to normality. Categorical variables were compared using Pearson’s chi-square test or Fisher’s exact test, as appropriate. Multivariable logistic regression analysis was performed using variables with *p* < 0.1 in the univariable analysis. All analyses were performed with IBM SPSS version 24.0.0.0 software (IBM Corporation, Armonk, NY, USA). A *p* values <0.05 were considered statistically significant.

## Results

### Predialysis demographic and clinical characteristics

The predialysis demographic and clinical characteristics are summarized in Table 1. The patients’ mean age was 61 ± 14 years, and 65.7% were men. DM and hypertension were found in 53.8 and 65.9% of the patients, respectively. The most common cause of kidney disease was diabetic nephropathy (45.0%). The mean BUN and BUN/Cr ratio were 39.5 ± 11.4 mg/dL and 15.1 ± 4.9 mg/mg, respectively.

**Table 1.** Baseline predialysis^a^ characteristics.

Characteristics	Total *N* = 453
eGFR (mL/min/1.73 m^2^)	21.9 ± 1.7
Age (years)	60.6 ± 14.1
Sex (male)	297 (65.7%)
BMI (kg/m^2^)	24.6 ± 3.8
Comorbidities	
Diabetes mellitus	243 (53.8%)
Hypertension	297 (65.9%)
Heart failure	32 (7.1%)
Ischemic heart disease	74 (16.4%)
Etiologies of renal disease	
Diabetic nephropathy	204 (45.0)
Hypertensive nephropathy	29 (6.4)
Chronic glomerulonephritis	109 (24.1)
Polycystic kidney disease	17 (3.8)
Others or unknown	94 (20.8)
Medications	
ACEi/ARB	369 (81.6%)
Loop diuretics	152 (33.6%)
Statin	300 (66.4%)
Laboratory findings	
BUN (mg/dL)	39.5 ± 11.4
Serum Cr (mg/dL)	2.69 ± 0.49
BUN/Cr ratio (mg/mg)	15.1 ± 4.9
Hemoglobin (g/dL)	11.2 ± 1.7
Serum albumin (g/dL)	3.9 ± 0.5
Serum uric acid (mg/dL)	7.4 ± 1.7
Total carbon dioxide (mmol/L)	21.1 ± 3.0
Urine PCR (g/g)	2.3 (1.2, 4.1)

Continuous variables are expressed as mean ± standard deviation or median (interquartile range), and categorical variables are expressed as number (percentage).

eGFR: estimated glomerular filtration rate; BMI: body mass index; ACEi/ARB: angiotensin-converting enzyme inhibitor/angiotensin receptor blocker; Cr: creatinine; BUN: blood urea nitrogen; PCR: protein to creatinine ratio.

^a^Predialysis was defined as the time point just before eGFR reached <20 mL/min/1.73 m^2^.

### Parameters at the time of dialysis initiation

**Figure 1.**
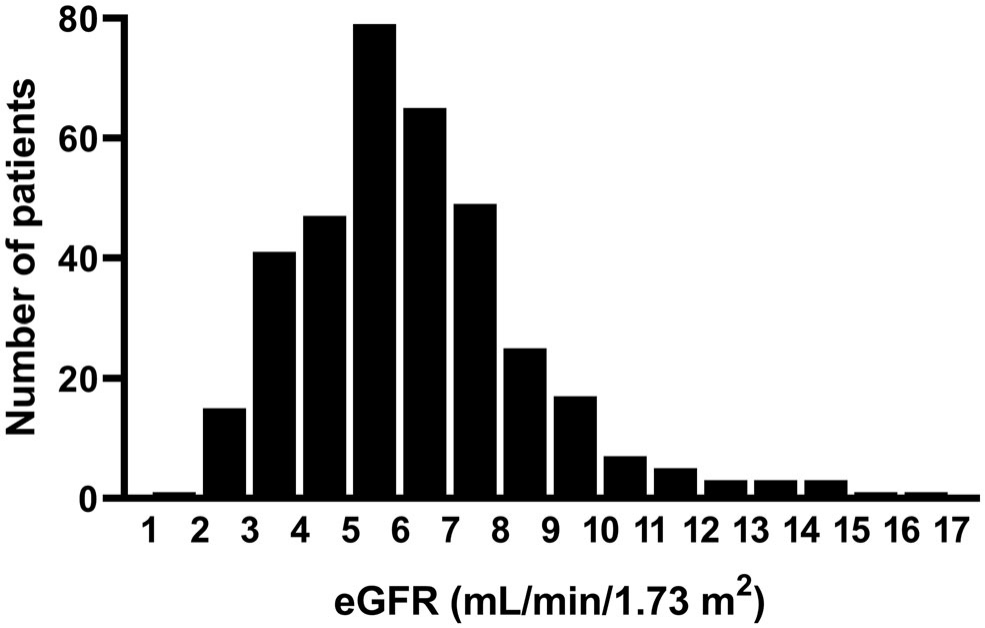
Distribution of eGFR at dialysis initiation. Overall, the median eGFR at dialysis initiation was 5.8 (4.6–7.3) mL/min/1.73 m^2^. Arbitrarily, we defined early initiation of dialysis as starting dialysis at eGFR ≥7.8 mL/min/1.73 m^2^ based on the upper 20% of eGFR at the initiation of dialysis. eGFR: estimated glomerular filtration rate.

Overall, the median eGFR was 5.8 (4.6–7.3) mL/min/1.73 m^2^ at the time of dialysis initiation. The distribution of eGFR at dialysis initiation is depicted in Figure 1. The highest quintile of eGFR at dialysis initiation was 7.8 mL/min/1.73 m^2^ and defined as early initiation. The early initiation group (*n* = 91) and the late dialysis group (*n* = 361) started dialysis at an eGFR of 9.1 (8.4–10.8) and 5.4 (4.2–6.4) mL/min/1.73 m^2^, respectively. The prevalence of heart failure at dialysis initiation increased in both groups compared to predialysis. Although not statistically significant, heart failure was more common in the early dialysis group than in the late dialysis group (24.2 vs. 15.7%, *p* = 0.058). Blood pressure at dialysis initiation was similar between two groups.

BUN at dialysis initiation was lower (81.9 ± 23.3 vs. 97.7 ± 29.9 mg/dL, *p* < 0.001), but the BUN/Cr ratio was higher (15.4 ± 5.1 vs. 11.0 ± 3.5 mg/mg, *p* < 0.001), in the early initiation group than in the late initiation group (Table 2). Serum albumin was lower in the early initiation group than in the late initiation group; however, the difference was only marginally significant (3.57 ± 0.67 vs. 3.72 ± 0.53 mg/dL, *p* = 0.050). Serum sodium and potassium level at dialysis initiation were comparable between the two groups. Total carbon dioxide and uric acid levels at dialysis initiation tended to be higher and lower in the early initiation group than the late initiation group, respectively, but values were not statistically significant (17.4 ± 2.1 vs 15.8 ± 4.0, *p* = 0.053 and 7.4 ± 2.1 vs 7.9 ± 2.3, *p* = 0.053, respectively). Serum corrected calcium was higher (8.5 ± 0.9 vs. 8.2 ± 1.0, *p* = 0.006) and phosphate (4.9 ± 1.1 vs. 6.0 ± 1.0, *p* < 0.001) and intact parathyroid hormone (PTH) [197 (102–272) vs. 268 (179–408), *p* < 0.001] were lower in the early initiation group than in the late initiation group. On the other hand, NT-proBNP level was not different between groups, but only 121 values were available for analysis.

**Table 2.** Parameters at dialysis initiation.

	Late initiation	Early initiation	*p* Value
	(*n* = 361)	(*n* = 91)	
eGFR (mL/min/1.73 m^2^)	5.4 (4.2–6.4)	9.1 (8.4–10.8)	NA
Heart failure	57 (15.7)	22 (24.2)	0.058
Systolic blood pressure (mmHg)	146 ± 19	145 ± 20	0.726
Diastolic blood pressure (mmHg)	78 ± 12	77 ± 12	0.465
BUN (mg/dL)	97.7 ± 29.9	81.9 ± 23.3	<0.001
Serum Cr (mg/dL)	9.1 ± 2.9	5.4 ± 1.1	<0.001
BUN/Cr ratio (mg/mg)	11.0 ± 3.5	15.4 ± 5.1	<0.001
Hemoglobin (g/dL)	8.9 ± 1.5	9.0 ± 1.3	0.485
Serum albumin (g/dL)	3.72 ± 0.53	3.57 ± 0.67	0.050
Uric acid (mg/dL)	7.9 ± 2.3	7.4 ± 2.1	0.053
Sodium (mmol/L)	137 ± 5	137 ± 5	0.194
Potassium (mmol/L)	4.8 ± 0.8	4.6 ± 0.8	0.809
Potassium >6.0 mmol/L	22 (6.1)	3 (3.3)	0.299
Total CO_2_ (mmol/L)	15.8 ± 4.0	17.4 ± 2.1	0.053
Total CO_2_ <10 mmol/L	20 (6.3)	3 (3.4)	0.435
Corrected calcium^a^ (mg/dL)	8.2 ± 1.0	8.5 ± 0.9	0.006
Phosphate (mg/dL)	6.0 ± 1.0	4.9 ± 1.1	<0.001
Intact PTH (412)^b^ (pg/mL)	268 (179–408)	197 (102–272)	<0.001
NT-proBNP (121)^b^ (pg/mL)	6862 (1912–19,646)	6659 (1546–15,867)	0.607

Variables are expressed as mean ± standard deviation or median (interquartile range).

Continuous variables were compared using an independent *t*-test or Mann-Whitney *U* test according to normality. Categorical variables were compared using Pearson’s chi-square test or Fisher’s exact test, as appropriate.

eGFR: estimated glomerular filtration rate; BUN: blood urea nitrogen; Cr: creatinine; CO_2_: carbon dioxide; NA: not applicable; NT-proBNP: N-terminal prohormone of brain natriuretic peptide; PTH: parathyroid hormone.

^a^Corrected calcium (mg/dL)=serum calcium (mg/dL) × 0.8 × (4–serum albumin [mg/dL]).

^b^Number of available data.

### Predictors of early initiation of dialysis

In univariable logistic regression analysis, DM, hypertension, heart failure, serum albumin <4.0 mg/dL, BUN/Cr ratio >15, and hyperuricemia were associated with early initiation of dialysis (Table 3). Among the causes of ESKD, chronic glomerulonephritis was associated with late initiation compared with diabetic nephropathy in the univariable model. In multivariable analysis, four factors maintained an independent association with early initiation: heart failure (OR 3.575, 95% CI 1.592–8.025, *p* = 0.002), serum albumin level <4.0 mg/dL (OR 2.227, 95% CI 1.303-3.773, *p* = 0.003), BUN/Cr ratio >15 mg/mg (OR 1.918, 95% CI 1.157–3.179, *p* = 0.012), and hyperuricemia (OR 1.842, 95% CI 1.049–3.234, *p* = 0.033). DM and the causes of kidney disease were not associated with early initiation in multivariable analysis.

**Table 3.** Predialysis^a^ predictors of early initiation of dialysis^b^.

	Univariable			Multivariable		
	OR	95% CI	*p* Value	OR	95% CI	*p* Value
Age (years)	1.013	0.996–1.031	0.132			
Sex (male)	1.479	0.890–2.458	0.131			
BMI (kg/m^2^)	0.987	0.928–1.050	0.683			
Diabetes mellitus	1.672	1.040–2.688	0.034	1.312	0.509–3.378	0.574
Hypertension	0.648	0.404–1.040	0.072	0.744	0.442–1.253	0.266
**Heart failure**	3.994	1.910–8.349	<0.001	**3.575**	**1.592–8.025**	**0.002**
Ischemic heart disease	1.343	0.745–2.421	0.327			
Etiology of renal disease			0.164			0.468
Diabetic nephropathy	ref			ref		
Hypertensive nephropathy	0.659	0.239–1.820	0.421	0.989	0.259–3.767	0.987
Chronic glomerulonephritis	0.466	0.244–0.890	0.021	0.638	0.225–1.812	0.399
Polycystic kidney disease	0.422	0.093–1.909	0.262	0.877	0.151–5.115	0.884
Others or unknown	0.910	0.580–1.629	0.751	1.372	0.502–3.748	0.538
Loop diuretics	1.029	0.633–1.672	0.908			
ACEi/ARB	0.971	0.538–1.752	0.921			
Statin	1.266	0.768–2.085	0.355			
Anemia^c^	0.667	0.392–1.135	0.135			
**Serum albumi*n* < 4.0 mg/dL**	0.524	0.326–0.842	0.008	**2.217**	**1.303–3.773**	**0.003**
**BUN/Cr ratio >15**	1.801	1.132–2.866	0.013	**1.918**	**1.157–3.179**	**0.012**
**Hyperuricemia**^d^	1.695	0.998–2.879	0.051	**1.842**	**1.049–3.234**	**0.033**
Urine PCR (g/g)	0.999	0.919–1.087	0.989			

Multivariable logistic regression analysis was conducted with variables with *p* < 0.1 in the univariable analysis. The independent predictors of early initiation of dialysis in bold type.

eGFR: estimated glomerular filtration rate; BMI: body mass index; ACEi/ARB: angiotensin-converting enzyme inhibitor/angiotensin II receptor blocker; BUN: blood urea nitrogen; Cr: creatinine; PCR: protein-to-creatinine ratio.

^a^Predialysis was defined as the time point just before eGFR reached <20 mL/min/1.73 m^2^.

^b^Early initiation of dialysis was defined as the initiation of dialysis at GFR ≥7.8 mL/min/1.73 m^2^.

^c^Anemia was defined as hemoglobin level <13.5 mg/dL in men and <12 mg/dL in women.

^d^Hyperuricemia was defined as serum uric acid level >7.0 mg/dL in men and >6.0 mg/dL in women.

### Unplanned dialysis and characteristics related to dialysis initiation

The proportion of patients who underwent unplanned dialysis was higher in the early initiation group than in the late initiation group (75.8 vs. 62.3%, *p* = 0.016) (Table 4). The median time from predialysis to dialysis initiation tended to be shorter in the early initiation group than in the late initiation group (765 [410–1658] vs. 973 [615–1567] days, *p* = 0.083). No differences in the rate of eGFR decline and in admission status at the time of dialysis initiation were observed between the two groups. Unplanned groups showed a higher eGFR decline rate than the planned group (6.4 [3.9–11.4] vs. 5.4 [2.9–8.8], *p* = 0.005).

**Table 4.** Pre-ESKD characteristics according to timing of dialysis initiation.

	Total	Late initiation^a^	Early initiation^a^	*p* Value
		(*n* = 361)	(*n* = 91)	
Unplanned dialysis^b^	294 (65.0%)	225 (62.3)	69 (75.8)	0.016
Interval to dialysis initiation (days)	945 (569–1599)	973 (615–1567)	765 (410–1658)	0.083
Rate of eGFR decline (/year)	6.1 (3.5–10.1)	6.11 (3.76–9.88)	5.78 (2.47–11.60)	0.142
Inpatient dialysis initiation^c^	356 (78.8%)	278 (77.0)	78 (85.7)	0.070

Continuous variables are median (interquartile range) and categorical variables are expressed as number (percentage).

Continuous variables were compared using Mann–Whitney *U*-test. Categorical variables were compared using Pearson’s chi-square test or Fisher’s exact test, as appropriate.

ESKD: end-stage kidney disease; eGFR: estimated glomerular filtration rate.

^a^Early initiation of dialysis and late initiation of dialysis were defined as the initiation of dialysis at GFR ≥7.8 mL/min/1.73 m^2^ and GFR <7.8 mL/min/1.73 m^2^, respectively.

^b^Unplanned dialysis was defined as not creating a permanent vascular access at least 1 month before the initiation of dialysis.

^c^Inpatient dialysis initiation was defined as initiation of dialysis during admission or in emergency room.

### Sensitivity analyses

We performed sensitivity analyses to confirm the robustness of the results. We repeated logistic regression analyses after excluding patients who underwent continuous renal placement therapy or received intensive care during the early period of RRT, or underwent living donor kidney transplantation within 30 days of dialysis initiation. The analyses also revealed that heart failure, low serum albumin level, high BUN/Cr ratio, and hyperuricemia were independent predictors of early initiation of dialysis.

## Discussion

To provide the best pre-ESKD care, we focused on identifying patients who required dialysis at a higher eGFR. Among those who received pre-ESKD nephrology care, the eGFR at the time of dialysis initiation was 5.8 (4.6–7.3) mL/min/1.73 m^2^, and 20% started dialysis at eGFR >7.8 mL/min/1.73 m^2^ (defined as early initiation of dialysis). Heart failure, low serum albumin, high BUN/Cr ratio, and hyperuricemia when the eGFR decreased to approximately 20 mL/min/1.73 m^2^ were independent predictors of early initiation of dialysis. These findings provide new insights suggesting that preparation of access as well as dialysis initiation can be determined in an individualized manner.

Timely preparation for RRT is crucial in pre-ESKD patients. If the preparation for RRT is delayed, patients are more likely to initiate dialysis in an unplanned manner and be exposed to the risk of complications related to the central vein catheter [13]. A smooth, planned transition to RRT is associated with better survival and quality of life [14,15]. Conversely, too early placement of vascular accesses may result in some accesses not being used until death, increased number of interventions to maintain vascular access patency before dialysis, or shortening the actually available lifespan of vascular accesses, especially with an arteriovenous graft [12,16,17]. Potential complications after vascular access creation, such as high-output heart failure, are additional concerns [18].

In this study, a considerable time lag (200 days) from the predialysis baseline to the start of dialysis was observed between the early and late initiation groups, although both groups showed remarkably similar eGFR decline rates. In other words, nephrologists need to consider the clinical characteristics associated with early initiation of dialysis, as well as the rate of eGFR decline, when preparing pre-ESKD patients for RRT. To date, few studies have focused on the predictors of dialysis initiation at a higher GFR. Clark et al. showed that old age, male sex, and various comorbidities, including coronary heart disease and DM, are associated with the initiation of dialysis at a higher eGFR [19]. In addition to comorbidities, our study evaluated the predialysis clinical characteristics just before reaching eGFR 20 mL/min/1.73 m^2^, including laboratory findings, and suggested that early preparations for RRT are recommended for patients with heart failure, low serum albumin, high BUN/Cr ratio, and hyperuricemia. Further clinical studies are needed to confirm these findings.

It is not clear when planning for RRT should begin during the course of CKD. The Kidney Disease: Improving Global Outcomes guideline does not suggest a specific GFR level, but recommends planning RRT when the risk of kidney failure is greater than 10–20% within one year, as measured using a risk prediction tool. The aim of this recommendation was to ensure that patients were referred to a specialist kidney care service for more than one year of dialysis [9]. Another guideline recommends that permanent vascular access should be established when the patient has an eGFR of 15–20 mL/min and has progressive kidney disease [10]. According to the results of our study, even in the early initiation group, it took more than median two years to initiate dialysis. These results suggest that it could be reasonable to decide whether to prepare for dialysis early or late at eGFR lower than 20 mL/min/1.73 m^2^, such as eGFR 15 mL/min/1.73 m^2^.

Heart failure was associated with a 4-fold increased likelihood of early dialysis initiation. Serum Cr-based eGFR values in patients with advanced heart failure are likely to overestimate the actual GFR probably owing to malnutrition [20,21]. Therefore, patients with heart failure may need to initiate dialysis at a lower actual GFR than serum Cr-based eGFR. In addition, patients with heart failure may need earlier initiation of dialysis because of their vulnerability to volume overload.

Low serum albumin level, high BUN/Cr ratio, and hyperuricemia were also independent predictors for early initiation of dialysis. We speculate that enhanced systemic inflammation and sarcopenia can be common mediators of these findings [22,23]. Interestingly, the BUN level at dialysis initiation was lower in the early initiation group than in the late initiation group. This suggests that dialysis was not initiated in advance solely because of the high BUN in the early initiation group. Conversely, the early initiation group may be vulnerable to uremic symptoms or complications even at low BUN levels.

As expected, patients who required dialysis at a higher eGFR more frequently underwent the first hemodialysis without a permanent vascular access (defined as unplanned dialysis). Several studies have demonstrated that unplanned dialysis is associated with higher mortality than planned dialysis [15,24–26]. Late referral to a nephrologist is one of the known risk factors of unplanned dialysis [26,27]. We excluded patients who received care from nephrologists for <6 months; however, unplanned dialysis was commonly observed overall and was more frequent in the early initiation group. Our results indicated that permanent vascular access needs to be created in advance in patients at a risk for early initiation of dialysis.

This study had some limitations. First, owing to the retrospective design, we were unable to exclude the influence of unmeasured confounders. Comprehensive chart review and sensitivity analyses were conducted in an attempt to reflect various aspects of patient characteristics. Second, this was a single-center study conducted in Korea and the initiation of dialysis is influenced by the attitudes of patients and physicians as well as various socioeconomic factors, including healthcare system. Therefore, absolute GFR values of this study cannot be applied to patients in different regions or countries. However, risk factors for initiating dialysis at a relatively high eGFR may be clinically useful regardless of absolute eGFR values. In addition, no other study has analyzed the risk factors for early initiation of dialysis at predialysis time points, such as at eGFR 20 mL/min/1.73 m^2^, and the results of this study do not contradict the known pathophysiology. Third, because most patients have been referred to local centers for maintenance dialysis, data on long-term outcomes were unavailable. Finally, data on the causes of initiating dialysis, such as uremia or hypervolemia, were unavailable for individual patients. It was difficult to clearly identify the reason for the initiation of dialysis using electronic medical records alone. However, hyperkalemia and metabolic acidosis were not severe in the early initiation group compared to the late initiation group. In addition, as mentioned earlier, the initiation of dialysis was probably determined by uremic symptoms or complications, given that the BUN level in the early initiation group was lower than that in the late initiation group, consistent with the assumptions of this study.

## Conclusions

Our study attempted to identify the predictors of early initiation of dialysis at a relatively high eGFR and found that heart failure, high BUN/Cr ratio, low serum albumin level, and hyperuricemia were associated with early initiation of dialysis. In patients at risk of early initiation of dialysis, clinicians can provide predialysis care earlier and consider early creation of vascular access. Further studies are needed to confirm whether the application of this policy can reduce unplanned dialysis.
